# Transmembrane protein topology prediction using support vector machines

**DOI:** 10.1186/1471-2105-10-159

**Published:** 2009-05-26

**Authors:** Timothy Nugent, David T Jones

**Affiliations:** 1 Bioinformatics Group, Department of Computer Science, University College London, Gower Street, London, WC1E 6BT, UK

## Abstract

**Background:**

Alpha-helical transmembrane (TM) proteins are involved in a wide range of important biological processes such as cell signaling, transport of membrane-impermeable molecules, cell-cell communication, cell recognition and cell adhesion. Many are also prime drug targets, and it has been estimated that more than half of all drugs currently on the market target membrane proteins. However, due to the experimental difficulties involved in obtaining high quality crystals, this class of protein is severely under-represented in structural databases. In the absence of structural data, sequence-based prediction methods allow TM protein topology to be investigated.

**Results:**

We present a support vector machine-based (SVM) TM protein topology predictor that integrates both signal peptide and re-entrant helix prediction, benchmarked with full cross-validation on a novel data set of 131 sequences with known crystal structures. The method achieves topology prediction accuracy of 89%, while signal peptides and re-entrant helices are predicted with 93% and 44% accuracy respectively. An additional SVM trained to discriminate between globular and TM proteins detected zero false positives, with a low false negative rate of 0.4%. We present the results of applying these tools to a number of complete genomes. Source code, data sets and a web server are freely available from .

**Conclusion:**

The high accuracy of TM topology prediction which includes detection of both signal peptides and re-entrant helices, combined with the ability to effectively discriminate between TM and globular proteins, make this method ideally suited to whole genome annotation of alpha-helical transmembrane proteins.

## Background

Alpha-helical transmembrane (TM) proteins constitute roughly 30% of a typical genome and are involved in a wide variety of important biological processes including cell signaling, transport of membrane-impermeable molecules and cell recognition. However, due to the experimental difficulties involved in obtaining high quality crystals, this class of protein is severely under represented in structural databases, making up only 1.7% of known structures in the PDB [1,2]. Given the biological and pharmacological importance of TM proteins, an understanding of their topology – the total number of TM helices, their boundaries and in/out orientation relative to the membrane – is essential for structural and functional analysis, and directing further experimental work. In the absence of structural data, sequence-based prediction methods allow TM protein topology to be investigated.

Early prediction methods, based on the physicochemical principle of a sliding window of hydrophobicity combined with the 'positive-inside' rule [3], have been superseded by machine learning approaches which prevail due to their statistical formulation. These include Hidden Markov models (HMMs), neural networks (NNs) and more recently, support vector machines (SVMs). While NNs and HMMs are capable of producing multiple outputs, SVMs are predominantly binary classifiers therefore multiple SVMs must be employed to classify the numerous residue preferences before being combined into a probabilistic framework. Like NNs and HMMs, SVMs are capable of learning complex relationships among the amino acids within a given window with which they are trained, particularly when provided with evolutionary information. However, they are considered more resilient to the problem of over-training compared to other machine learning methods.

One problem faced by modern topology predictors is the discrimination between TM helices and other features composed largely of hydrophobic residues. These include targeting motifs such as signal peptides and signal anchors, amphipathic helices, and re-entrant helices – membrane penetrating helices that enter and exit the membrane on the same side, common in many ion channel families. The high similarity between such features and the hydrophobic profile of a TM helix frequently leads to crosstalk between the different types of predictions. Should these elements be predicted as TM helices, the ensuing topology prediction is likely to be corrupted. Some prediction methods, such as SignalP [4] and TargetP [5], are effective in identifying signal peptides, and may be used as a pre-filter prior to analysis using a TM topology predictor. Phobius [6] uses a HMM to successfully address the problem of signal peptides in TM protein topology prediction, while PolyPhobius [7] further increases accuracy by including homology information. Other methods such as TMLOOP [8], TOP-MOD [9] and OCTOPUS [10] have attempted to identify re-entrant regions, the latter two in combination with a TM topology predictor, but there is significant room for improvement.

TM topology predictors also exist that are able to use experimentally derived information in order to guide topology prediction. With reliable experimental data, prediction accuracy is likely to benefit substantially. Methods include HMM-TM [11], HMMTOP [12] and TMHMMfix [13]. Tools such as SOSUI [14] and PRED-CLASS [15] are designed to discriminate between globular and TM proteins, while others such as PRED-TMBB [16] specialise in the discrimination and prediction of beta-barrel TM proteins.

A key element when constructing any prediction method is the use of a high quality data set for both training and validation purposes. Previously described TM data sets such as the Möller set [17] have contained relatively few sequences with structures available (33), but substantially more with TM region annotation based on varying types of biochemical characterisation (166). A number of experimental methods, including glycosylation analysis, insertion tags, antibody studies and fusion protein constructs, allow the topological location of a region to be identified. However, such studies are often conflicting [18,19] and also risk upsetting the natural topology by altering the protein sequence. As a result, orientation and helix boundary errors in databases are not infrequent and add an element of noise. While such noise is often well tolerated by machine learning methods, the problem is more significant in smaller data sets. With a view to unifying, updating and verifying existing resources while minimising topological error, a new TM data set has been created containing protein sequences with topologies derived solely from crystal structures.

We thus present a new TM topology predictor trained and benchmarked with full cross-validation on a novel data set of 131 sequences with crystal structures. The method uses evolutionary information and four SVMs, combining the outputs using a dynamic programming algorithm, to return a list of predicted topologies ranked by overall likelihood, and incorporates signal peptide and re-entrant helix prediction. Overall, the method predicted the correct topology and location of TM helices for 89% of the test set, a significant improvement on our previous NN-based method MEMSAT3 [20]. An additional SVM has been trained to discriminate between TM and globular proteins with zero false positives and a low false negative rate of 0.4%, making this method highly suitable for whole genome analysis.

## Results and discussion

### Support vector machine performance

Table 1 shows the per residue performance of each of the five SVMs used by the method. The TM helix/¬TM helix SVM performs significantly better than the re-entrant helix/¬re-entrant helix and inside loop/outside loop SVMs, and slightly better than the signal peptide/¬signal peptide and TM protein/globular protein SVMs, reflecting the relative ease with which the hydrophobic signal of a TM helix is detected compared to sequence features within the other topological regions. The Matthews correlation coefficient (MCC) value of 0.80 compares favourably with the equivalent value of 0.76 achieved by MEMSAT3 using a NN when cross-validated against the same test set. We found that the inclusion of unlabelled data for transductive learning led to a slightly lower MCC of 0.77, in addition to increasing training time, and thus parameter optimisation time, substantially. As a result we excluded unlabelled data when training the final model.

**Table 1:** SVM per residue accuracy.

SVM	Window size	Kernel	MCC
TM Helix/¬TM Helix	33	RBF	0.80
Inside Loop/Outside Loop	35	Polynomial*	0.63
Re-entrant Helix/¬Re-entrant Helix	27	RBF	0.34
Signal Peptide/¬Signal Peptide	27	RBF	0.76
TM Protein/Globular Protein	33	RBF	0.78

Column 2: Window size – the size of the sliding window in residues. Column 3: Kernel – SVM kernel type. RBF = radial basis function. Column 4: MCC – Matthews correlation coefficient. * The Inside Loop/Outside Loop SVM was trained using a third-order polynomial kernel.

The inside loop/outside loop SVM was the only SVM to perform optimally using a polynomial kernel, which justifies our use of multiple SVMs to classify each of the residue preferences rather than a single multiclass ranking SVM. The highest MCC value we could achieve using a radial basis function (RBF) kernel for this SVM was 0.35, significantly lower than the value of 0.63 achieved using a third-order polynomial kernel, therefore demonstrating that no single kernel function is capable of optimally separating all the data classes and suggesting the structure of loop data is strongly favoured by this kernel.

Detection of re-entrant helices remains challenging compared to other regions, with lack of training data a significant issue. Despite the addition of 64 proteins to the training set, all were homologous to one of the original 11 re-entrant helix-containing proteins and were therefore removed from the respective training files. Their contribution was therefore reflected by a low false positive rate of 0.008, but a low true positive rate of 0.478 owing to the lack of positive training examples.

In contrast, the signal peptide/¬signal peptide and TM protein/globular protein SVM performance was close to that of the TM helix/¬TM helix SVM, aided by sufficient quantities of training data. While largely driven by hydrophobicity, the signal peptide/¬signal peptide SVM must accurately discriminate between signal peptides, which contain a 7–15 residue long hydrophobic helix, and an equally hydrophobic but slightly longer TM helix. For all signal peptide-containing proteins, the residues ranked highest by the SVM appear to be close to the C-terminal end of the signal peptide region, suggesting the SVM is efficiently detecting the polar and uncharged 3–8 amino acid residue long C-region and the neutral residues that lie adjacent to the cleavage point [21]. Similarly, the TM protein/globular protein SVM must discriminate between hydrophobic residues that compose TM helices and those that form the core of globular proteins, a challenge reflected by the difference in MCC compared to the TM helix/¬TM helix SVM.

### Overall topology prediction accuracy

Table 2 shows the overall topology prediction accuracy when applying the method to the test set of 131 TM proteins, alongside results for a number of other recent topology predictors. MEMSAT-SVM and MEMSAT3 results are fully cross-validated as described above, with all proteins homologous to the target being removed from training sets, while results for the remaining methods were obtained from their respective web servers and consequently are not cross-validated. OCTOPUS was also trained exclusively using proteins with crystal structures available, of which 121 sequences (92%) are present in the test set, therefore results are likely to be significantly overestimated.

**Table 2:** Benchmark results for the SVM-based method ('MEMSAT-SVM') against a selection of leading topology predictors

Method	Algorithm	Correct helix count	Correct helix locations	Correct N-terminal	FP helix	FN helix	Correct SP topology	Correct RE topology	Correct topology
MEMSAT-SVM	SVM	95%	91%	91%	4%	5%	93%	64%	89%
OCTOPUS	NN + HMM	86%	83%	84%	14%	2%	21%	73%	79%
MEMSAT3	NN	84%	76%	84%	8%	8%	57%	64%	76%
ENSEMBLE	NN + HMM	77%	76%	79%	18%	5%	7%	55%	67%
PHOBIUS	HMM	75%	76%	79%	9%	16%	93%	36%	63%
HMMTOP	HMM	77%	76%	78%	18%	6%	29%	64%	63%
PRODIV	HMM	79%	64%	76%	19%	8%	0%	18%	57%
SVMTOP	SVM	66%	64%	66%	22%	22%	0%	55%	53%
TMHMM	HMM	75%	68%	72%	14%	20%	29%	55%	53%
PHDhtm	NN	75%	54%	55%	23%	30%	29%	18%	45%

Column 1: Method – Prediction method. Column 2: Algorithm – Underlying machine-learning algorithm. Column 3: Correct helix count – Fraction of sequences with the correct number of TM helices predicted. Column 4: Correct helix locations – Fraction of sequences with the correct number and locations of TM helices predicted. Column 5: Correct N-terminal – Fraction of sequences with the correct N-terminal location predicted. Column 6: FP helix – Fraction of sequences with at least one over predicted TM helix. Column 7: FN helix – Fraction of sequences with at least one under predicted TM helix. Column 8: Correct SP topology: Fraction of sequences that contain signal peptides that have correct overall topology predicted. Column 9: Correct RE topology: Fraction of sequences that contain re-entrant helices that have correct overall topology predicted. Column 10: Correct topology: Fraction of sequences that have correct overall topology predicted, requiring the correct number and location of TM helices and correct location of the N-terminal. TM helices must overlap their defined positions by at least 5 residues.

To assess overall topology prediction accuracy, correct prediction of 3 components were required: the N-terminal location, number of TM helices and TM helix locations, based on an overlap of at least 5 residues with boundary definitions. Correct signal peptide and re-entrant helix predictions were not required for a correct overall topology prediction, though failure to predict these features was likely to result in an incorrect topology. Based on this definition, MEMSAT-SVM correctly predicts topology in 89% (116 out of 131) of cases, a 10% improvement on OCTOPUS which predicted 79% (103) of cases correctly (column 10). Using a more stringent criterion of a 10-residue helix overlap, the margin increases to 11% (MEMSAT-SVM 87%, OCTOPUS 76%), suggesting good segment end point prediction. In terms of the 3 individual components, MEMSAT-SVM is consistently better than all other methods (columns 3–5), and in particular performs well at predicting the correct number of TM helices (95% accuracy). MEMSAT-SVM also had a balanced number of over- and under predictions (columns 6–7) which is favourable to avoid bias towards either type of prediction, and suggests good sensitivity while avoiding over predicting helices. Since this work was completed, an extension to the OCTOPUS method which incorporates signal peptide prediction, SPOCTOPUS [22], has been released. This method achieved 87% accuracy on the test set, largely addressing the poor performance of OCTOPUS on sequences containing signal peptides (column 8).

### Signal peptide and re-entrant helix prediction

MEMSAT-SVM correctly predicts the topology of 93% (13 out of 14) of proteins which contain signal peptides, a substantial improvement on the limited signal peptide prediction capability of our previous method MEMSAT3. In all 13 cases, signal peptides were also predicted. This accuracy is matched by PHOBIUS, the only other method that is specially trained to identify signal peptides in TM proteins. Amongst proteins that did not contain signal peptides, no false positive signal peptides were predicted. Proteins containing re-entrant helices proved much harder to predict, with only 64% (7 out of 11) correctly predicted. This is matched by MEMSAT3 and HMMTOP, though is slightly lower than the 73% (8) accuracy achieved by OCTOPUS. However, this additional correct prediction could well be attributed to the overlap between the test and training sets, as, in the absence of cross-validation, MEMSAT-SVM is able to predict 82% (9) topologies correctly. In terms of predicting re-entrant helices, MEMSAT-SVM identifies 44% (8 out of 18) with 2 false positive predictions, which compares favourably with OCTOPUS results of 22% (4) with 4 false positives. Since the numbers of proteins containing re-entrant helices and signal peptides are relatively small (14 and 11 respectively), care should be taken when interpreting these results as a relatively large percentage difference in performance may only reflect the correct prediction of one additional sequence.

### Erroneous predictions

MEMSAT-SVM incorrectly predicts topologies in 15 cases. Four of these correspond to proteins containing re-entrant helices that are erroneously predicted as TM helices – ABC transporter BtuCD, Proton Glutamate Symport protein, Aquaporin Z and Clc chloride channel (PDB: 1l7vB, 1xfhA, 2abmH and 2feeB) – accounting for the majority of over predicted TM helices. The remaining over prediction is due to a highly hydrophobic N-terminal region within a chain from Cytochrome bc1 (PDB: 1sqxD).

In seven cases, incorrect topologies are a result of under predicted TM helices – Photosystem I (chains A, L and K), Steryl-sulfatase, Light-Harvesting Complex II, Particulate Methane Monooxygenase and Sodium/proton antiporter 1 (PDB: 1jb0A, 1jb0L, 1jb0K, 1p49A, 1vcrA, 1yewB and 1zcdB). These under predictions fall into two categories; weakly predicted helices (PDB: 1jb0A, 1jb0L, 1jb0K, 1p49A and 1vcrA) or prediction of one helix rather than two shorter ones (PDB: 1yewB and 1zcdB). Of the weakly predicted helix errors, sequence analysis indicates low hydrophobicity for many of these helices, often due to a large fraction of charged residues. Such helices are therefore extremely difficult to predict and suggest a novel membrane insertion mechanism. Other helices appear sufficiently hydrophobic to be detected; errors are possibly the results of PSI-BLAST alignment which reduce their detectability.

The remaining three incorrect predictions are all single TM helix proteins that are inverted – Photosystem I, Cytochrome bc1 and Cytochrome b6f (PDB: 1jb0I, 1p84I and 1q90N). In all three cases, the confidence of the prediction compared to the correct topology (measured by the difference between the two scores) is extremely small. With no clear signal to differentiate between either orientation, interplay with other chains from the same protein may influence the final conformation.

### Prediction accuracy using the Möller and TOPDB data sets

We additionally tested prediction performance using a subset of 184 sequences from the Möller set, described in [11,14], composed of sequences annotated using both crystal structures and biochemical characterisation (Table 3). The Möller set consists of a significantly higher fraction of eukaryotic sequences compared to the data set described above. TM protein crystallisation techniques usually involve over expression hosts, such as *Escherichia coli*, which to date have worked mainly for prokaryotic TM proteins since eukaryotic TM proteins are still very difficult to over express [23]. Crystal structure-based sets, while providing more accurate TM helix boundary definitions, thus suffer from this bias towards prokaryotic sequences, so methods trained exclusively using such data sets run the risk of performing poorly when predicting the topologies of eukaryotic sequences. Based on recent updates to SWISS-PROT annotations and under full cross-validation, MEMSAT-SVM achieved 78% accuracy and MEMSAT3 achieved 77%. In the absence of cross-validation, SPOCTOPUS also achieved 77% accuracy, with OCTOPUS the next best method scoring 69%. This performance suggests MEMSAT-SVM offers robust prediction accuracy on proteins from both eukaryotic and prokaryotic domains.

**Table 3:** Prediction performance using the Möller and TOPDB data sets

Method	Möller	TOPDB
MEMSAT-SVM	78%	67%
OCTOPUS	69%	64%
MEMSAT3	77%	66%
ENSEMBLE	61%	51%
PHOBIUS	67%	62%
HMMTOP	64%	57%
PRODIV	46%	37%
SVMTOP	70%	42%
TMHMM	60%	56%
PHDhtm	45%	49%

Column 1: Prediction method. Column 2: Results using the Möller data set. Column 3: Results using the TOPDB data set.

We then tested performance using the TOPDB [24] data set, a comprehensive collection of TM protein containing experimentally derived topology information (Table 3). It currently contains records for 1452 alpha-helical TM proteins. Using this data set, MEMSAT-SVM achieved 67% accuracy, MEMSAT3 66%, OCTOPUS 64% and PHOBIUS 62%. The data set also contains 317 sequences containing signal peptides. Of these, MEMSAT-SVM correctly predicted the topologies for 77% of cases. This value was lower than that of PHOBIUS which achieved 85% accuracy. However, the MEMSAT-SVM false positive rate for signal peptide prediction is 7%, half the PHOBIUS value of 14%. These results show that on this data set, MEMSAT-SVM signal peptide performance is below that of PHOBIUS, though MEMSAT-SVM overall prediction accuracy is 5% higher due to the relatively poor performance of PHOBIUS on sequences that do not contain signal peptides (a substantially larger fraction) – 54% accuracy compared to 63% for MEMSAT-SVM. These results should again be treated with caution as they were not cross-validated.

These results are clearly lower than those attained using the crystal structure-based data set, and we believe this is likely due to errors in TOPDB. We analysed sequences from the original, uncorrected Möller set that at the time did not have crystal structures. 55 of these sequences now have a homologous PDB structure (E-value < 0.001), and of these only 38 (69%) of the original Möller topologies are correct based on current OPM definitions (taking into account only the N-terminal location and TM helix count). There is no reason to believe that the error rate in other data sets such as TOPDB, composed predominantly of sequences whose topologies were determined by biochemical means, should be significantly different. Perfect prediction methods are therefore unlikely to be able to achieve results higher than this, while older methods trained on erroneous topologies have the potential to achieve higher scores but may in reality be poorer predictors, a fact likely to be highlighted when tested against a crystal structure-based set.

### Discriminating between globular and transmembrane proteins

Using the combined set of 2453 test cases, we assessed performance in discriminating between globular and TM proteins (Table 4). As a discrimination threshold, a number of residues were required to be predicted as part of a TM helix by the SVM in order to classify the protein as TM. This threshold was adjusted in order to minimise the margin between the false positive (FP) and false negative (FN) rates, therefore avoiding bias towards either type of prediction. A 0% FP rate and 0.4% FN rate was achieved using only a single residue as the threshold, an improvement on the MEMSAT3 neural network-based approach (0.5% FP, 0.5% FN) and SOSUI (0.3% FP, 1.1% FN). OCTOPUS matched the FP rate but achieved a higher FN rate, while PHOBIUS matched the FN rate but achieved a higher FP rate. These low error rates suggest that MEMSAT-SVM is extremely well suited to whole genome analysis.

**Table 4:** Results for TM/globular protein discrimination rates.

Method	Algorithm	False positive rate	False negative rate
MEMSAT-SVM	SVM	0.00%	0.44%
MEMSAT3	NN	0.50%	0.50%
SOSUI	Hydrophobicity analysis	0.33%	1.10%
OCTOPUS	NN + HMM	0.00%	2.51%
PHOBIUS	HMM	2.72%	0.44%

### Application to a number of complete genomes

Table 5 shows the results of applying the TM/globular predictor to a number of complete genomes. We estimate that a typical genome contains between 24% and 33% TM proteins, which is slightly higher than previous estimates of between 20% and 30% [32]. Two organisms that have a noticeably higher fraction of TM proteins are *Caenorhabditis elegans *and *Takifugu rubripes*. *Takifugu rubripes *is known to have extensive channel heterogeneity compared to *Homo sapiens*, with 10 *Homo sapiens *voltage-gated calcium channel α1-subunit genes revealing 21 orthologous genes in *Takifugu rubripes*. Phylogenetic analysis reveals that this is due to fish lineage specific α1-subunit subtype duplication [33]. Similar increased subtype diversity has also been detected in the appetite receptor neuropeptide Y GPCR family that may have arisen as a result of ray-finned fish tetraploidization [34]. *Caenorhabditis elegans *is known to have an exceptionally large number of 7 TM receptors and rhodopsin-like membrane proteins [35], thought to have been arisen through duplication events, that possibly imply functional relations between homologous 7 TM domains [36]. *Escherichia coli *has the lowest fraction of TM proteins of all the species we analysed, which may be a consequence of the lack of internal membrane systems in prokaryotes [37].

**Table 5:** The fraction of proteins predicted as transmembrane, and to contain re-entrant helices and signal peptides, in a number of complete genomes.

Species	Fraction of genome predicted as TM proteins	Fraction of TM proteins predicted to contain re-entrant helices	Fraction of TM proteins predicted to contain signal peptides
Caenorhabditis elegans	33%	2%	33%
Canis familiaris	31%	2%	27%
Danio rerio	29%	2%	26%
Drosophila melanogaster	27%	2%	33%
Escherichia coli	24%	2%	28%
Homo sapiens	26%	2%	35%
Mus musculus	29%	2%	30%
Pan troglodytes	26%	2%	33%
Takifugu rubripes	33%	3%	26%
Xenopus tropicalis	31%	2%	23%

We then carried out full topology prediction on sequences predicted to be TM proteins and analysed these for the presence of re-entrant helices and signal peptides. In most species, re-entrant helices were detected in at least 2% of TM proteins, with more than 3% detected in *Takifugu rubripes *which can be explained by the extensive channel heterogeneity discussed above. However, given the low true positive rate of 44%, this figure is likely to be an underestimate. A positive predictive value (PPV) of 0.8 suggests a value in the range 3–4.5% is more realistic. This range is close to one previous estimate of 5% [38] but below another of 10% [8], although the latter was based on a broader definition of re-entrant regions that did not necessarily contain helical secondary structure.

**Figure 1:**
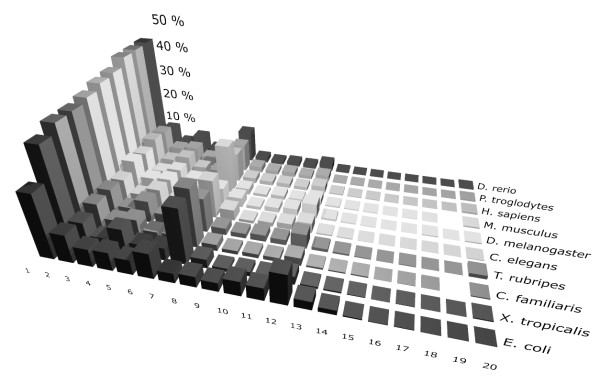
**Topology prediction results for a number of complete genomes**. X-axis: Number of predicted TM helices. Y-axis: Fraction of all predicted TM proteins. Z-axis: Species.

Topology prediction results illustrate consistent trends across all species, with significant peaks at 7 TM helices representing GPCRs (in eukaryotes) and 12 TM helices representing transporters proteins (Figure 1). A slight preference for even-numbered topologies (excluding GPCRs) can be explained by the formation of 2 helix hairpins as independent units during protein assembly, therefore favouring topologies with even numbers of TM helices [39]. In all species, the most dominant topology is a single TM helix. These results are consistent with previous studies [40].

## Conclusion

In this paper we have implemented a novel SVM-based TM protein topology predictor, an area previously dominated by HMM and NN-based machine learning approaches, and have shown that it outperforms a selection of the best performing prediction methods when fully cross-validated on a novel high resolution data set of 131 protein sequences. This data set includes proteins containing both re-entrant helices and signal peptides, features that this method is also able to predict. The method has also been benchmarked on the Möller data set, which contains a higher fraction of eukaryotic sequences, improving on the best current methods. And we have achieved extremely low false positive and false negative rates for TM/globular protein discrimination. Using these tools, we have estimated the fraction of TM proteins, re-entrant helices and signal peptides in a number of complete genomes. Overall, our results suggest that MEMSAT-SVM is ideally suited to whole genome annotation of alpha-helical TM proteins.

## Methods

### Assembling a novel data set of transmembrane proteins

The novel data set was based solely on crystal structure data. Additional information was collected from MPTOPO [41], OPM [42], PDB_TM [43], SWISS-PROT [44] and from the literature. SWISS-PROT files were parsed for entries containing the keyword 'TRANSMEM' in feature table (FT) lines. N-terminal data was also extracted using keyword 'TOPO_DOM' where available. To avoid partial sequences being included, any entries containing keywords such as 'FRAGMENT' were excluded. Sequences were then scanned against the PDB in order to identify entries for which the TM region had complete structural coverage. Alignments occasionally highlighted chain breaks. In these cases, the sequence was excluded unless a visual inspection ensured the topology could not be cast in doubt by the break. This left a redundant data set containing 944 sequences which was then homology reduced at the 40% sequence identity level. A number of sequences were then removed. These included colicins (e.g. PDB: 1col) and bee venom (PDB: 2mlt) which are bilayer disrupting and thus are not native integral membrane proteins, sequences labelled as 'secreted protein', and sequences where the N-terminal location or topology could not be verified.

OPM was then used to define TM helix boundaries, or in the absence of an OPM entry, PDB_TM was used. OPM uses a theoretical multi-feature approach to position proteins in a membrane which has been shown to be in good agreement with experimental studies of 24 TM proteins. In some cases where a visual inspection appeared to indicate an incorrect placement of the membrane, PDB_TM helix boundary definitions were used instead. For example, OPM lists Mechanosensitive channel protein MscS (PDB: 2oau) as having two TM helices, neither of which fully cross the membrane, whereas the PDB_TM definition of 3 TM helices appears more plausible.

PDB_TM was also used to annotate proteins containing re-entrant helices. A re-entrant helix was defined as a helix-containing region that enters and exits the membrane on the same side, penetrating at least 6Å but not more than 6Å from the opposite membrane face. Re-entrant regions that did not contain a helix formed by at least three contiguous amino acid residues were excluded. Sequences containing signal peptides were then labelled according to SWISS-PROT annotations.

The composition of the final data set containing 131 sequences, all with available crystal structures, verifiable topology and N-terminal locations, is show in table 6.

**Table 6:** Data set composition.

Protein class	Number in set
Prokaryotic	92
Eukaryotic	37
Viral	2
	
Single-spanning TM segment	57
Multiple-spanning TM segments	74
	
Contains re-entrant helix	11
Contains signal peptide	14
	
Total	131

### Support vector machine training

As Support Vector Machines (SVMs) are binary classifiers [45], we chose to combine multiple SVMs to classify each of the residue preferences found in TM proteins. Although multiclass ranking SVMs do exist, they are generally considered unreliable since in many cases no single mathematical function exists to separate all classes of data from one another [46]. We therefore trained four SVMs to classify TM helix/¬TM helix, inside loop/outside loop, re-entrant helix/¬re-entrant helix and signal peptide/¬signal peptide. Residue labelling was performed according to our data set definitions.

PSI-BLAST [47] was used to generate position-specific scoring matrices for each of the proteins in the data set using the UniRef 90 database. Two iterations were performed with a profile-inclusion E-value threshold of 0.001 in order to reduce false positive hits, to which TM proteins are more prone than globular proteins [48]. For each residue in a sequence, a sliding window approach was used to create a feature vector of length 20 × W, where W is the size of the window centred on the target residue. Where the window extended beyond the protein termini, empty feature values were set to zero. All values for each feature position where then normalised by Z-score to enable faster SVM convergence. Initial attempts at scaling values between 0 and 1 had resulted in lower overall prediction accuracy.



x is the raw score to be normalised. μ and σ are the mean and standard deviation PSI-BLAST score  for each of the 20 amino acid, generated using profiles for all 131 sequences.

In order to accentuate the contribution of re-entrant helices for which data is particularly sparse, the sequences of 64 proteins, all homologous to the 11 re-entrant helix-containing sequences in our initial data set, were also used to train the TM helix/¬TM helix and re-entrant helix/¬re-entrant helix SVMs. Helix, loop and re-entrant helix boundaries were determined by PDB_TM definitions.

We also attempted to train the TM helix/¬TM helix SVM using unlabelled data via transduction. In transduction, the learning task is to assign labels to unlabelled data as accurately as possible [49]. SVMs can perform transduction by finding the hyperplane that maximises the margin relative to both the labelled and unlabelled data, in order to improve the generalisation performance. We selected sequences from SWISS-PROT identified by the MEMSAT3 TM/globular protein discriminator as TM proteins. Sequences with greater than 40% sequence identity to sequences in the labelled data set were removed, as were those with signal peptides predicted by SignalP. Of those remaining, 135 sequences were used as unlabelled training data.

For training the signal peptide/¬signal peptide SVM, we included data from the Phobius training set which contains 2654 well annotated examples of TM and globular proteins, with and without signal peptides. This was supplemented by a search of SWISS-PROT for sequences labelled with the keyword 'SIGNAL' (but excluding entries labelled 'POTENTIAL' or 'BY SIMILARITY') to add to the signal peptide set, and sequences without keyword 'SIGNAL' to add to the non-signal peptide set. The combined set was then homology reduced at the 40% sequence identity level, leaving 3205 (1222 with signal peptides, 1983 without signal peptides) sequences for which PSI-BLAST profiles were then generated as outlined above.

Stringent cross validation was performed using a jack knife test (leave-one-out cross validation) for the TM helix/¬TM helix, inside loop/outside loop and re-entrant helix/¬re-entrant helix SVMs. In training, the target sequence, along with any other sequences with greater than 25% sequence identity, were excluded. For the signal peptide/¬signal peptide SVM we used 10-fold cross validation, again excluding sequences from the training set with greater than 25% sequence identity to any sequence in the test set. For training and classification, SVM-Light [50] was used. The performance of several kernels was investigated in combination with a comprehensive grid search of SVM parameters. To determine optimal windows sizes, the data set was split randomly into two and the highest scoring window which ranked equally in each split was selected, therefore demonstrating consistency between data sets and reducing the risk of overfitting. We used the MCC to optimise these values which is a more robust measure than using recall or precision alone [51].

To calculate a list of topologies ranked by overall likelihood, the TM helix/¬TM helix, inside loop/outside loop and signal peptide/¬signal peptide raw SVM outputs were combined in a modified version of the dynamic programming algorithm used in the original MEMSAT method [52]. The algorithm was simplified by treating TM helices as discrete units, rather than separating them into inside, outside and middle components, though a signal peptide state was added. Loop regions between predicted TM helices were scanned for re-entrant helices using the re-entrant helix/¬re-entrant helix raw SVM output and a simple scoring function. For evaluating signal peptide preference, residues with positive signal peptide scores up to position 40 in a target sequence were added to the outside loop score and subtracted from the inside loops score where positive, in order to direct prediction towards a non-cytoplasmic amino-terminus. The value was also scaled by a factor of 10 and subtracted from the TM helix SVM score to prevent TM helix prediction. Residues were therefore predicted to lie in one of five different topological regions: inside loop, outside loop, TM helix, re-entrant helix and signal peptide.

To evaluate performance, four metrics were used. Firstly, correct location of the amino terminus; secondly, correct number of TM helices; thirdly, correct number and location of TM helices (based on an overlap of at least five residues with the helix boundaries in our data set) and fourthly, correct overall topology. For comparison, we also evaluated a number of other leading topology predictors. For this method and MEMSAT3, the appropriate cross-validated training data was used in assessing performance. Where equivalent data was unavailable for the other methods, performance is likely to be overestimated as it is likely that there is significant overlap between test and training sets. We also assessed performance of the method against proteins containing signal peptides and re-entrant helices.

We also trained an additional SVM to discriminate between TM and globular proteins, to be used as a pre-filter prior to TM topology prediction. For SVM training, we used the data set of 131 TM proteins and 416 globular proteins from non-redundant PDB chains as used by MEMSAT3. To accurately compare with MEMSAT3 we used exactly the same test set consisting of 184 TM proteins from the Möller data set and a separate set of 2269 non-redundant globular protein chains, giving a total of 2453 test cases. PSI-BLAST profiles were generated for all sequences and 10-fold cross validation was used to assess performance, again removing sequences from the training fold with greater than 25% sequences identity to any sequence in the test fold.

For whole genome analysis, ten genomes – nine eukaryotic and one prokaryotic – were downloaded from the Ensembl [53] and NCBI [54] websites. Protein sequences were extracted and PSI-BLAST profiles were generated using the SWISS-PROT database. The TM/globular predictor was used to identify TM proteins, which were then subject to full topology prediction.

## Availability

MEMSAT-SVM is available as downloadable source code and as a web server from the URL below and is free for non-commercial use. All data sets are also available, and cross-validation SVM model files are available on request. The software has been tested on a Linux operating system. In order to compile and run, the gcc compiler, Perl interpreter, and NCBI tools are required.



## Competing interests

The authors declare that they have no competing interests.

## Authors' contributions

Both authors have contributed equally to this work. Original source code was developed by DTJ. This was re-written and extended by TN. DTJ provided direction for computational aspects of the algorithm and biological/biophysical insight into aspects of membrane protein structure. Manuscript was prepared by TN and has been read and approved by DTJ.
